# The Micro-Organisms of the Mouth and Their Association with Disease

**Published:** 1889-09

**Authors:** Geo. W. Watson


ARTICLE IV.

### THE MICRO-ORGANISMS OF THE MOUTH AND THEIR ASSOCIATION WITH DISEASE.

(Read before Odonto-Chirurgical Society of Scotland, by Geo. W. Watson.)

After describing the principal characteristics of bacteria and touching upon their distribution, the reader proceeded to give Cohn's classification as follows:—

1. Sphærobacteria, globules, micrococci.
2. Microbacteria, short rods, bacterium.
3. Desmobacteria, long rods, bacillus vibrio.
4. Spirobacteria, spirals, spirochaeti, and spirillum.

To these might be added—Mucoidei, mould; saccharomycetes, yeasts.

The methods of propagation, cultivation, &c., having been briefly referred to, Mr. Watson went on to say:—I shall now endeavor to show you, and describe, by means of lantern transparencies taken from my negatives, some of the different organisms which I have cultivated from the mouth, and also some slides taken from negatives lent me by my friend, Dr. Edington, whose kindness and help I hereby acknowledge.

Dr. Miller, of Berlin, separated out and described some twenty-five different organisms from the human mouth.

I shall commence with the organisms of caries:—

*Slide* 1—Leptothrix buccalis growing, from piece of carious dentine L, is a very common organism in the mouth. In caries it is found growing from the external surface of the tubules, and does not penetrate into the tubules until they are completely softened and broken up.

*Slide* 2—There are several different varieties of L, and this is one more slender and with longer filaments than that of buccalis, leptothrix gigantea, long rods, short rods, found in the mouths of dogs, sheep, cats, and other animals. Cruikshank, in his "Bacteriology," says that leptothrix is found in the mouth in the form of long rods, short rods, cocci, and spirilla, which assertion I very much doubt, more especially as this opinion is taken from some of the works of the older writers. It is very difficult to get a good growth of leptothrix buccalis for examination, but I hope to be able to do so, and report at some future time.

*Slide* 3—This slide represents very well chalky, carious enamel. Along with the broken up enamel prisms there are a few filaments of leptothrix, but no other organisms are present, which accords with what Dr. Miller says in regard to this.

*Slide* 4—Organisms grown from deepest layer of carious dentine. With a sterilized instrument the outer layers of carious tissue was rapidly removed till nearly sound tissue was reached. Some of this was put into a tube of nutrient jelly, and incubated on the third day. The tube was found swarming with leptothrix and micrococci, with liquefaction of jelly, degeneration of growth taking place on second week. As liquefaction of the jelly goes on the organisms all fall to the bottom of liquid area.

*Slide* 5—Micrococcus pure culture separated from above.

*Slide* 6—Section of carious dentine exhibiting tubules packed and distended with micro-organisms.

*Slide* 7—Same, more highly magnified. The organisms which penetrate the tubules are principally micrococci, though sometimes bacilli and spirilla are found, but never leptothrix. Before micro-organisms can gain admission to the tubules decalcification must have taken place, and this is readily accomplished at weak points of the teeth by means of the numerous ferment organisms found in the mouth.

I quite agreed with Dr. Miller that there are areas of softened non-infected dentine which contain no organisms.

Dr. Miller, of Berlin, in his article on dental caries, says that he has been able to produce this condition artificially on sections of sound teeth put into beef extract and sugar, and inoculated with a pure culture of the organisms from caries. The probability is that this is quite correct, as Dr. Miller has done very good and thorough work in the investigation of the organisms of the mouth; at the same time his experiments have not been confirmed by other investigators. Whenever I can find time and opportunity I hope to take this subject up. There is not the slightest doubt that dental caries can to a considerable extent be kept down by keeping the mouth in a thoroughly clean and aseptic condition—but I shall refer to this further on.

*Slide* 8—Bacteria grown from scrapings of tongue.

*Slide* 9—Two epithelial scales from tongue covered with the same organisms, in chains and singly.

*Slide* 10, 11, 12, 13—This series of slides shows well the variety of micro-organisms found in mouths where no attention to cleanliness is paid. Bacilli, micrococci, bacteria, spirilla, and leptothrix are all present.

*Slide* 14—Colony of bacilla cultivated from pus taken from root of tooth.

*Slide* 15—Streptococcus pyogenes.—This is the special organism of suppuration, inoculation with this organism causing the development of an abscess. Improperly cleaned instruments, you can therefore understand, might be productive in the mouth of severe abscess if used on other patients—or more serious results. Streptococcus erysipelas, also pathogenic.

*Slide* 16—Saccharomyces albicans or oidium albicans.

*Slide* 17—One of the yeasts is the organism associated with thrush in the mouth of infants or debilitated subjects.

The mucous membrane is covered with greyish white patches, consisting of epithelial cells, this fungus, and the mycelia of moulds. Spore formation is well shown in one of the slides. The preparation from which this photo-micrograph was taken was obtained from the throat of a patient suffering from phthisis. Associated with ulcerative stomatitis, there is found a bacillus, which seems to have a great deal to do with the spreading of the disease in calves, rabbits, and mice. It usually proves fatal, owing to the rapid development of the bacillus invading all the important organs.

After showing several other slides illustrating organisms associated with various diseases, the paper was concluded in the following words:—

The presence in the mouth of such numbers of microbes, many of which are pathogenic, suggests to us the possibility of infection from such organisms after tooth extraction by means of the open wounds, and may account for some of those cases of obscure inflammatory after-trouble which we sometimes come across. I make a point of recommending all patients, after the removal of several teeth, to use a powerful antiseptic mouth-wash in view of this. More particularly should this be attended to in the case of debilitated subjects with a tubercular tendency, as the open wounds would form a good medium for the development and growth of tubercle bacilli. Gangrene of the lungs, chronic pyæmia, abscesses of throat and mouth, &c., have by various authors been ascribed to the action of the micro-organisms of the mouth. Dr. Miller mentions that he has been able to produce septicæmia in rabbits and mice, by injecting into the lung saliva from the mouth of a perfectly healthy person. This, of course, is due to the organisms micrococci, &c., in the saliva, or to some of their products, ptomaines. To keep the mouth in a thoroughly healthy condition, all roots and teeth that cannot be filled should be removed, as they form sources of infection, and the teeth should be thoroughly brushed with a tooth-powder having antacid and antiseptic properties. Just before going to bed is perhaps the best time to do this, as during sleep the mouth is at rest, and fermentation in active progress, especially between the teeth where food is most likely to lodge. By the diligent use of antiseptics, and silk threads passed between the teeth, the mouth can be kept comparatively free from microbes.

The point to consider now is, which antiseptic is best for our purpose? Mercuric chloride is by many considered the best antiseptic, but for use in the mouth it is both very poisonous and disagreeable, as well as not being very stable. When mercuric chloride, especially strong solutions, comes in contact with albumen it unites with the sulphur to form mercuric sulphide, which is perfectly useless as an antiseptic. As for carbolic acid, it has been experimentally proved to be too feeble and insufficient, as it takes one or two days for a five per cent. solution of carbolic to destroy the spores of anthrax.

Dry heat is a valuable antiseptic, and is extremely useful in the treatment of teeth with putrid canals when immediate root-filling is carried out.

Dr. Edington has investigated the antiseptic germicidal and bacteriacidal properties of hydronapthol, one of the coal-tar derivatives. This he has done very thoroughly, with the result that it proves to be the most valuable antiseptic we have. It is soluble in cold water in the proportion of 1 in 1100, which is stronger than 1.1000 mercuric chloride. It is soluble in hot water in the proportion of 1 in 300, and mixes in any proportion with glycerine. It is not poisonous, has a pleasant odor, and can be used full strength, if necessary, without any bad result. I have been using a 10 per cent. solution of hydronapthol for some little time for the treatment of putrid conditions, &c., and find it better than any antiseptic yet tried. The proprietors are making up, specially for dental purposes, tooth powder containing 5 per cent. of the antiseptic, a mouth-wash, a 10 per cent. solution, and cotton wool, specimens of which I hand around for inspection.—*London Dental Record.*

